# Prospect Theory: A Bibliometric and Systematic Review in the Categories of Psychology in Web of Science

**DOI:** 10.3390/healthcare10102098

**Published:** 2022-10-20

**Authors:** Júlia Gisbert-Pérez, Manuel Martí-Vilar, Francisco González-Sala

**Affiliations:** 1 Departamento de Psicología Básica, Universitat de València, Avgda. Blasco Ibañez 21, 46010 Valencia, Spain; 2 Departamento de Psicología Evolutiva y de la Educación, Universitat de València, Avgda. Blasco Ibañez 21, 46010 Valencia, Spain

**Keywords:** prospect theory, applied psychology, health, decision making, behavior, prevention

## Abstract

Prospect Theory (PT) is an alternative, dynamic explanation of the phenomenon of risky decision making. This research presents an overview of PT’s history in health fields, including advancements, limitations, and bibliometric data. A systematic and bibliometric review of the scientific literature included in the psychological categories of Web of Science (WoS) was performed following the PRISMA 2020 statement for systematic reviews. A total of 37 studies (10 non-empirical and 27 empirical) were included in the sample. Bibliometric results showed thematic variability and heterogeneity regarding the production, researchers, and methodologies that are used to study PT. The systematic results highlight three main fields of PT research: preventive and screening behaviors, promotion of healthy habits, and COVID-related decision making. Personal and contextual factors which alter the usual pattern specified by PT are also described. To conclude, PT currently has an interdisciplinary character suitable for health promotion, with recent studies broadening its applicability.

## 1. Introduction

Decision making under risk has been a subject of social research for several centuries. This extensive scientific interest has allowed the development of a large theoretical and experimental body on decision making under risky conditions [1], leading to new models that have attempted to solve problems such as the excessive emphasis on normativity. This paper highlights the contribution of Prospect Theory (PT).

PT was created by Kahneman and Tversky [2,3]. It developed as an alternative explanation of risky decision-making processes to Expected Utility Theory [4]. PT contemplates the presence of heuristics and limitations in human cognition, which result in biases and deviations from what is considered normative. However, these deviations are considered systematic and could be studied to improve decision making [5].

PT is based on two fundamentals. The first points out that, in deciding between the different choice options, we depend on a frame of reference and not so much on the absolute value of the options, which violates the economic conception of rationality. The second foundation of the theory is loss aversion bias. Loss aversion refers to a greater sensitivity to potential losses than to potential gains of equal magnitude [5].

To justify these assumptions, controlled experiments were developed in which participants had to choose between different alternatives (usually two) with different probabilities of achieving certain outcomes [2,6]. The obtained results showed that the decision process comprised two phases, the editing phase and the evaluation phase. First, a reference point was set and the possible outcomes were framed as benefits or losses. The process ends with a personal assessment of the usefulness of the options [2,7]. Among the basic findings and principles of Kahneman and Tversky’s theory [2,3], the S-shaped value function, the four-fold pattern of risk preferences, the “probability weighting function”, the uncertainty effect, and “the reflection effect” are worth mentioning.

PT is a descriptive theory of human behavior which does not explain how people should theoretically make their decisions, but how they actually do [8]. It has been applied, not without difficulties, to different contexts, such as economics [5,9] and politics [10,11,12]. Likewise, its assumptions have been analyzed in more specific conditions, such as energy efficiency investment [13], terrorism [14], political participation [15], or climate policies [16].

One of Kahneman and Tversky’s key insights was that the way risky decisions are framed influences what is selected, and it does so in a way captured by the assumption of an S-shaped value function defined on changes from the status quo [2,17]. Health decisions inherently involve risky choices [18]. Thus, consistent with what PT predicts, subsequent work demonstrated that the way in which health information is framed (to focus on potential gains (e.g., benefits of healthy behavior) versus losses (e.g., harms of unhealthy behavior)) systematically influences decisions and choices [17,19]. In addition, the COVID pandemic also involved risky decision making at the societal level. Consistent also with PT, gain- or loss-framing of health information influenced decision making, and risk-free behaviors may be promoted [20]. 

In addition to the framing effect, alterations in the expected pattern of loss aversion have also been studied. Regarding PT in the psychological field, its application in substance addictions stands out for its inherent risky decision making. [21]. According to PT, low levels of loss aversion increase the likelihood of engaging in addictive behaviors. Drug users have been found to show lower loss aversion than non-users [21]. All of this can be taken into account by healthcare personnel to understand the resistance and ambivalence in the decision-making processes in consumer patients.

Given its long-standing interest and applicability, the aim of this study is to conduct a bibliometric and systematic review of the PT literature in health settings within the psychology categories of Web of Science (WoS), in order to provide an overview of the usefulness, applicability and limitations of the theory within this scientific discipline. This will allow the creation of a new resource pool from which replications of previous studies, scientifically argued critiques, or even new experiments or theories can emerge, leading to more critical and informed scientific developments. It may also help psychology and health professionals to understand human cognitive issues and promote good health.

## 2. Materials and Methods

A systematic and bibliometric review of the scientific literature of Prospect Theory [2,3] in the main WoS database was conducted. A protocol was registered in PROSPERO, with identification code CRD42022348325. The search was conducted in September 2022 following PRISMA 2020 statement for systematic reviews [22]. SPSS 22 statistical package, R package Bibliometrix [23] and WoS analysis were used for the bibliometric review. 

### 2.1. Information Sources and Search Strategy

A search was performed in the Web of Science database (Core Collection) with the search term “prospect theory” and “health”. Other databases were not consulted due to the number of studies identified and the objective of exploring the WoS psychology categories.

### 2.2. Eligibility Criteria and Selection Process

In the systematic search, the inclusion criteria were (a) containing the term “prospect theory” and “health” in topic, (b) being a scientific article, (c) being included in one of the psychological WoS categories: “behavioral sciences”, “neurosciences”, “psychology”, “psychology applied”, “psychology biological”, “psychology clinical”, “psychology educational”, “psychology experimental”, “psychology mathematical”, “psychology multidisciplinary”, “developmental psychology”, “psychology psychoanalysis”, or “psychology social”, and (d) being written in English or Spanish. 

**Figure 1:**
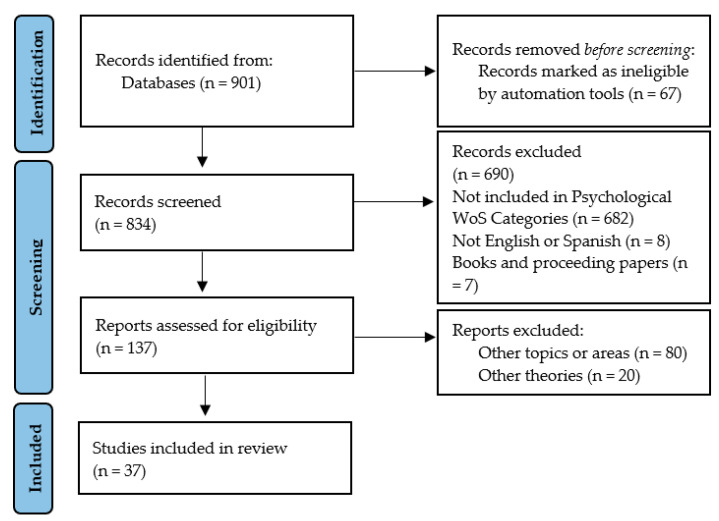
Flowchart of the selection and screening process of the systematic review articles according to the PRISMA method.

The exclusion criteria consisted o” (a)’addressing other topics (n = 80), (b) articles on other theories (n = 20), and (c) articles that were book chapters (n = 7). The selection and screening process is shown in Figure 1.

The selection process was performed by two investigators independently and then combined to reach a consensus. A third investigator supervised the results to confirm the quality of their work. 

### 2.3. Data Extraction

After the selection and analysis process, the final sample contained 37 articles. 

For the bibliometric review, the following variables were considered: year of publication, number of authors, distribution by country and continent, university affiliations, areas of research in psychology according to WoS, scientific journals, and key concepts. To perform the keyword co-occurrence networks), not all the terms were included, eliminating isolated nodes. For the systematic review, the following variables were considered: authors, year of publication, type of study, and main objective. For the empirical studies, we also extracted information on the sample, the methodology, the existence of a control group, and the main results and limitations. The bibliometric data extraction process was carried out using the WoS indicators, while data extraction for the systematic review was performed in the same way as the study selection process.

## 3. Results

### 3.1. Results of Bibliometric Review

**Figure 2:**
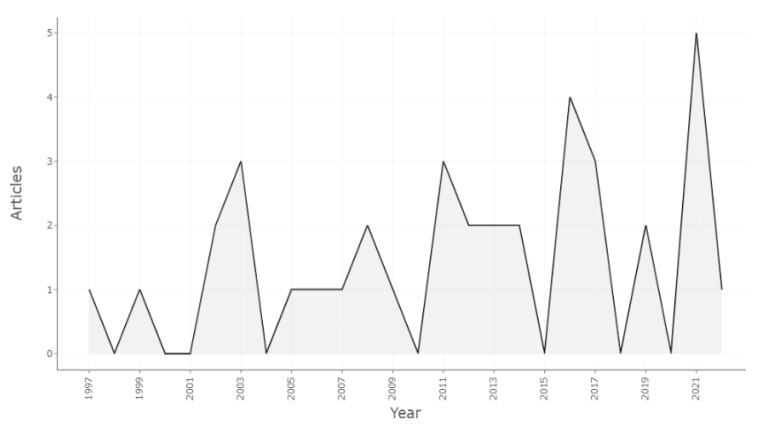
Annual scientific production.

Regarding TP production in the health field, the Figure 2 presents an irregular and increasing distribution with the highest production peak in 2021. In this year, several of the publications focused on the study and promotion of health behaviors in the COVID pandemic. The interest in applying PT to the field of health seems to have started in 1997, 18 years after the original study [2], indicating that the initial interests of this theory were focused on other fields. The last decade (2012–2022) accumulates 57% of the publications, highlighting the growing interest.

The sample includes 112 authors. Mainly, the contribution of P. Salovey (Yale University) to the field of health in PT (4 publications) stands out, followed by G. J. De Bruijn (University of Amsterdam) and A. J. Rothman (University of Minnesota System) (3 publications). The rest of the authors contribute in 2 (12% of authors) or 1 publication (86%). 

**Figure 3:**
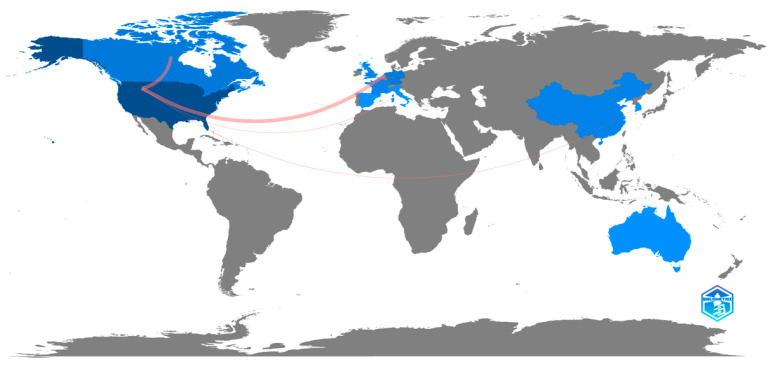
Country scientific production.

Figure 3 shows the distribution of scientific production by country, considering both internal (CMI) and international (CCM) collaborations. The sample included 12 countries in the Americas, Europe, Asia, and Oceania, and a total of 142 related publications. The USA had 81 linked publications (57%), followed by the Netherlands (16; 11%) and Canada, China, and Germany (7; 5%). Accordingly, the top five universities with the highest affiliations are Yale University (4), Maastricht University (3), University of Amsterdam (3), University of Minnesota System (3), and University of Minnesota Twin Cities (3). 

With 5 or fewer linked publications are Singapore, the UK, Australia, and France (4), Italy (3), South Korea (2), and Spain (1). Regarding international collaborations, the USA stands out with Canada and the Netherlands with two collaborations, followed by Germany–Spain–Netherlands, USA–Italy, and USA–South Korea with one collaboration.

Regarding the WoS psychology categories, the areas that appeared to be most linked to PT and health are Multidisciplinary Psychology (13 publications), Clinical Psychology (10), Psychology and Social Psychology (8), Applied Psychology (3), Experimental Psychology and Behavioral Sciences (2), and Developmental Psychology and Neurosciences (1). Among the categories that did not belong to Psychology, Economics and Public Environmental Occupational Health (2) and Gerontology, Hospitality Leisure Sport Tourism, Management, Nutrition Dietetics, Oncology, Psychiatry, Social Sciences, and Biomedical and Sport Sciences (1) stood out (Table A1).

A total of 27 scientific journals present articles related to PT and health. The scientific journals with the highest number of articles on PT in the area of health are the *British Journal of Health Psychology*, *Health Psychology*, and *Journal of Applied Social Psychology* (3, respectively). *Journal of Behavioral Medicine, Journal of Economic Psychology, Psychology Health and Social*, and *Personality Psychology Compass* have two publications each. The remaining 20 have only one publication.

**Figure 4:**
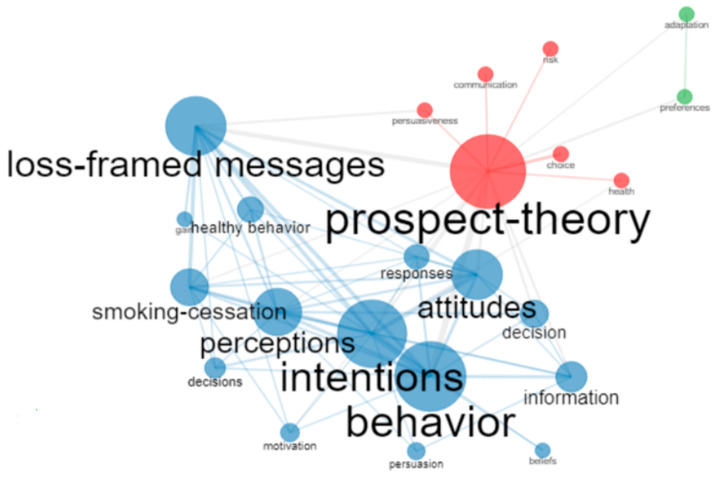
Co-occurrence network.

By analyzing the keyword co-occurrence networks, a general picture of the predominant terms in the study of PT and health was obtained. Figure 4 shows that “prospect-theory” was the term with the highest intermediation, i.e., presenting the highest number of links to other keywords. The other terms with the least intermediation were “intentions”, “loss-framed messages”, “behavior”, “perceptions”, “information”, and “attitudes.” The size of each block indicates the frequency of occurrence as an intermediate word. The figure shows three groups of keywords (blue, red, and green) and a closer link (by the thickness of the link) between “prospect-theory-behavior” and “behavior-intentions”.

### 3.2. Results of Systematic Review

Table A1 and Table A2 in Appendix A (non-empirical studies and empirical studies, respectively) show a synthesis of the data from the studies in the sample. Ten non-empirical studies (published between 1997 and 2021) and twenty-seven empirical studies (published between 1999 and 2022) were found. 

#### 3.2.1. Prospective Theory and Health Care Field

According to DeStasio [18], there are three main contributions of PT to the health domain. First, PT indicates that people will act differently depending on whether a situation is gain- or loss-framed compared to some reference point. Second, the reference point may have a particular impact on preventive health behaviors that are unpleasant themselves (e.g., vaccinations or invasive screening tests), where the risk of the immediate negative outcome (e.g., pain) is felt higher than the risk of the potential long-term outcome. Third, PT predicts that reframing health outcomes with respect to certainty would change decisions about health behaviors (as there is often an overweighting certainty). 

PT assumes that people respond predictably to potential gains and losses. They are risk-seeking when confronted with information about losses, but risk-averse when confronted with information about gains [19]. Thus, in the health field, gain-frames may be more beneficial to promote preventive behaviors, as well as loss-frames to favor detection behaviors [24]. One possible explanation is that prevention behaviors are perceived as low risk, while detection behaviors are perceived as high risk [19,25,26]. 

There have been many examples of successful use of PT in modeling decision making in health care settings. For example, it has been used to model health behaviors such as disease treatment [26], disease prevention [27,28,29,30,31], and encouraging altruistic behaviors such as egg donation [32]. On the one hand, Fridman et al. [26] investigated the relationship between physicians’ gain-loss recommendations and prostate cancer patients’ treatment choices. Results showed that physicians’ use of loss-related words correlated with recommendations for cancer treatment, and loss words were associated with patients’ choice of treatment. On the other hand, similar results to those hypothesized by PT were obtained in disease prevention studies, but variables have been found to influence the framing effect such as cultural differences [28] and credibility of the result [30]. However, having family members with the disease to prevent did not influence decision making [31]. 

Another focus of PT study has been life attitudes in healthy and sick patients and reference point [33,34,35,36,37]. Current health status determines one’s reference point. The reference point for an advanced cancer patient with a short life expectancy will be closer to death compared to an older adult with many years of expected survival. Thus, ill patients would prefer prolonging their life over quality of life, as was found in the results [33,34,36]. Likewise, sick patients rated a mild and a severe disease situation very differently, but healthy patients rated the two scenarios as much more similar [35]. In addition, having an overly pessimistic view of old age (e.g., not correctly predicting one’s own ability to adapt to the health problems of old age) may produce a self-fulfilling prophecy, showing reduced sensitivity to loss and impacting their health behaviors (e.g., underinvesting in future health) [37].

Given PT usefulness, public health (PH) agencies could perhaps benefit from utilizing PT in a way that would optimize the effectiveness of PH messaging to increase overall local and global adherence [24]. On the one hand, expectations and disappointment regarding health may influence happiness. A practical implication would be that doctors exaggerate the risk of bad health outcomes in the future, and emphasize that patients could not have prevented bad current outcomes [38]. On the other hand, the differences in reference point in healthy and sick people can be applied to the promotion of care or insurance plans, considering the preferences of both groups [33]. Lastly, depending on the intention to prevent or treat, gain-loss frameworks can be applied to achieve attitudinal and behavioral changes [24].

Despite all the potentialities of PT, it also has weaknesses. For instance, Van’t Riet’s review [39] includes studies of framing in the health care setting with contradictory results [39,40]. Therefore, it is necessary to carry out precise analyses of the subtle differences in the messages that may influence the receptors’ reactions. 

It should be noted that in decision making, it is important to consider variables beyond framing and risk. Among the studies reviewed, personality aspects such as psychopathy, ambivalence (e.g., persistence of attitudes, resistance to change), impulsivity, anxiety, or health involvement stand out [41,42,43]. Overall, personality characteristics of the respondents played a more important role as predictors of risk choices mainly in the negative frame [42,43]. Likewise, with individuals with high ambivalence, a greater persuasion appears with a negative framing (and vice versa), due to a possible negativity bias [41].

#### 3.2.2. Prospect Theory on Promoting Healthy Habits

PT has also been used to promote health-related attitudes and behaviors, which may reduce the occurrence of diseases. In the study of the framing effects on health issues, gain frames generally had an advantage over loss frames in promoting preventive behaviors (e.g., physical activity) [44,45]. Gallagher and Updegraff [44] concluded that “how a health message is framed is an important consideration in designing messages that promote preventive behaviors’’. In this regard, a gain message was associated with better semantic and affective evaluations of the message, but also a prime/frame and frame/source valence match was found more persuasive [45]. Hence, semantic consistencies must be taken into account, as they moderate the influence of message framing.

Therefore, it makes sense that, in the case of health-affirming behaviors such as physical activity (PA), messages framed around gains (i.e., benefits) rather than losses (i.e., costs) are often more effective [19,45,46]. PT has been applied through framed messages to promote PA [47], as well as the use of fitness apps [48]. The results of these studies showed an advantage of gain-framed messages in promoting sport intentions and attitudes, self-efficacy and sport practice itself [46,48]. Likewise, the effects of the framed PA messages were studied across all age and sex groups, demonstrating that older men may especially benefit from PA messages due to a possible age-related positivity effect [47]. 

In this context, although the gain frame in PA promotion is often more effective, it is important to consider the motivations associated with PA behavior and how the frame fits with these motivations [44]. All of this implies that the effect of framed messages is not simply based on the function of detection or prevention, but that personal motivations and interpretations must be considered. In addition, a possible interaction between source credibility and frame should be considered, as the gain frame together with a credible source (e.g., a physician) indicated higher exercise intentions and behaviors [49]. 

PT has also been applied to the promotion of healthy eating. On the one hand, PT predicted that the perceived positive value (i.e., benefit) associated with accumulating gains grows in an asymptotic, rather than linear, function [2]. This function applied to healthy intake suggests that less health gain may be associated with eating more pieces of fruit, and consequently, after having eaten a piece of fruit, individuals may see less value in eating more. This hypothesis was somewhat supported; health benefits that people assign to consuming increasing amounts of fruit appear to increase, but only if consumption of a variety of fruits throughout the day is considered [50]. On the other hand, the effect of autonomy on framing effects and fruit and vegetable consumption has been studied. Churchill and Pavey [51] observed that gain-framed messages only boosted fruit and vegetable consumption among those with high levels of autonomy; therefore, autonomy moderated the framing effect.

This gain-framing effect on preventive behaviors was also present in the use of sunscreen. Individuals who read gain-framed messages compared to the loss-framed ones were more likely to ask, repeatedly apply, and use sunscreen at the beach [52]. At the neural level, these results are consistent with greater activation of the medial prefrontal cortex (MPFC) to gain-framed messages. Higher MPFC activation reliably predicts subsequent behavior [53].

Moreover, in this sense, de Bruijn [54] explored the message framing effects to promote dental health using mouth rinse for 2 weeks. Their results coincided with the promotion of preventive actions, the gain-framed information to emphasize the preventive use of mouthwash being more appropriate. No framing effects were found in the detection conditions.

Frame effect on tobacco smoking cessation has also been studied [55]. Through messages framed in gain and loss and images illustrating positive and negative consequences, it was found that the intention to quit smoking was greater when negative images (e.g., unhealthy mouths) appeared, as well as when pictures of healthy mouths illustrated the presence of preventive action. On a practical level (e.g., health campaigns), the use of fear appealing communications with vivid negative images is one way to reduce tobacco use.

#### 3.2.3. PT, COVID Pandemic, and Social Behaviors

Understanding framing effects in PH messaging is important for improving adherence, and it is particularly important when considering messaging where loss of life can be avoided, such as during the COVID-19 pandemic [24]. PT has been used to study risky decision making and the promotion of behaviors to reduce virus transmission, such as physical distancing or vaccination. People’s behavioral response to a health crisis depends on how they perceive threat and their level of risk tolerance. Through PT, public health messages can be framed to influence adherence to health recommendations, taking into account other factors that may affect adherence.

Doerfler et al. [56] focused on risky decision making during the pandemic and its relationship with Dark Triad traits. Their results coincided with those presented by Tversky and Kahneman [57]. In a gain scenario (lives saved), individuals were more likely to opt for the certain option, thereby displaying a bias toward risk-aversion. In a loss scenario (lives lost), individuals were more likely to take greater risks.

During the COVID pandemic, maintaining an adequate physical safety distance was necessary to prevent the spread of the virus, especially indoors. Neumer et al.‘s [58] online and field experiment with manipulated gain- or loss-framed messages showed that loss-framed messages were more effective than gain-framed ones promoting physical distancing. The loss-frame advantage suggests that uncertainty about the true effectiveness of distancing to avoid contracting COVID-19 is high and that people are more willing to accept this uncertainty when faced with a potential loss than gain.

Another behavior studied since PT has been vaccination during the pandemic. Vaccination is an important tool to end pandemics, but the majority of the public must be willing to be vaccinated to reach herd immunity. Using health message framing, Reinhardt and Rossman [43] conducted an online experiment with framed messages with younger and older samples. Loss frames lead to significantly more positive vaccination attitudes in younger adults than gain frames, which affects their vaccination intentions. However, the effects of gain- and loss-framed messages on vaccination attitudes and intentions in older adults did not differ significantly. This difference was explained by an age-related positivity effect in the older sample, since they ignored the negatively framed information in the loss frame condition and focused on the positive ones.

Finally, some moderators studied in relation to the framing of health message interventions during pandemics have been respondents’ age, targeted beneficiaries (self or community), uncertainty (as mentioned above), loss-framing reactance, and personality traits as psychopathy, as mentioned above [24,43,56]. In relation to health, the age of respondents may imply differences in framing effects for variables such as positivity in older people [43]. Furthermore, greater persuasion has been found when messages are directed at the respondents themselves as opposed to the general community. Reactance is directly associated with attitudes and behaviors and is expressed in negative cognitions and emotions; therefore, it may result in more negative attitudes towards the promoted behavior [56]. Lastly, psychopathy emerged as the significant predictor of risk taking during the COVID-19 crisis.

## 4. Discussion

PT is a theory that attempts to explain dynamic changes in decision making, including aspects ignored by rational choice theories and highlighting the importance of situation and value in decision making [59]. In this study, a systematic review and bibliometric analysis of the literature on PT and health-related fields included in the WoS psychology categories was performed. The results of the bibliometric analysis have shown a growing international interest in the application of PT in health issues. The USA, followed by the Netherlands and Canada, have contributed the largest amount of literature on PT in health care settings. The analysis of the co-occurrence networks showed that the most frequent terms were prospect theory, intentions, behavior, and loss-framed messages, indicating the main interests of the application of PT in health. 

Regarding the results of the systematic review, heterogeneity has been found in the topics, methodology, and even in some results. The application of PT in health has mostly focused on the framing effects to promote health behaviors and the importance of people’s reference point. On the one hand, it has generally proven useful to use a gain frame to promote preventive health behaviors, whereas a loss frame seems to be more useful for treatment or detection behaviors. Therefore, when decision making involves low risk, gain-framed messages may be more effective, as well as loss framed messages in high-risk decisions. On the other hand, current health status is a key factor in decision making, as it determines the personal reference point. Current health status can influence the choice of future treatments or preferences about longevity or quality of life. 

Other areas in relation to health that have been studied in PT have been the promotion of healthy habits. PT has been shown to be useful in promoting healthy habits, using gain-framing primarily. These behaviors, in turn, can be preventive, thus promoting wellness. Furthermore, the COVID pandemic situation has allowed numerous applications of PT in an intrinsically risky and uncertain context, especially in the promotion of preventive behaviors (e.g., social distancing, vaccination).

In summary, from a PT perspective, it is possible to encourage certain health-related behaviors depending on the framing, decision risk, and variables that may influence decision making. It is important to note that some results have been shown to be contradictory, thus requiring an analysis of the choices to be balanced, as well as consideration of variables that may influence decision making (e.g., personality traits, certainty of sources, cultural differences, age). All this can be taken into account when developing preventive or screening programs, as well as to promote healthy behaviors, considering the particularities of the targeted social sector (e.g., healthy or sick people).

In conclusion, this systematic and bibliometric review provides interdisciplinary evidence of the functionality of PT for the study of decision making under risk, highlighting both PT basis and factors that modify the expected decision patterns. Although these factors can be considered to hinder the applicability of PT, knowing its limitations can be very beneficial in extending the theory to new fronts. Understanding cognitive aspects such as decision making is essential in fields such as psychology and health, as it allows planning better assessments and interventions to promote well-being.

### 4.1. Limitations

The present study has several limitations. First, including only articles in the sample limits the complete knowledge of the study topic. Second, due to the size of the sample and the interest in the psychological categories of the WoS, other databases were not consulted. This meant that only studies categorized within the areas of psychology in WoS were included, thus providing a bibliometric and systematic approach limited to this area, which explains why studies such as the original [2] are not included in the sample. The interest in the psychological fields and PT lies in the importance of the cognitive part in decision making and its importance as a health science. Third, aspects such as the sampling method or the method of information extraction have not been considered because little information was provided in the articles in the sample.

### 4.2. Future Directions

First, a study of similar characteristics is proposed in other fields to broaden the study of PT. Second, it is proposed to conduct empirical studies that apply PT to specific fields or problems related to cognitive aspects or decision making within psychology and health, such as behavioral addictions. Third, it would be interesting to continue with the study of variables that alter the patterns expected by PT in order to extend the scientific knowledge. In this way, a more complete scientific framework would be obtained and the scope of the theory itself would be broadened. Fourth, it would be very useful to create a training program for health care and health professionals to promote preventive health behaviors and treatment. In this line, it would be interesting to test the applicability of PT with minors, in order to promote healthy habits in early ages.

## Data Availability

The raw data supporting the conclusions of this article will be made available by the authors, without undue reservation.

## Appendix A

**Table A1:** Non-empirical articles included in the systematic review.

Year	Author	Main Aim	Methodology
2021	Edwards [24]	Literature from behavioral economic, heuristic, and behavioral analysis in relation to explaining how cognitive biases in public health messaging, and how best to improve the effectiveness of PH messages	Review
2021	Kocas [32]	To apply PT and the anchoring heuristic to demonstrate how donors’ initial exposure to information during recruitment, as well as the way in which risk is framed throughout, may influence their perception and decision making	Theoretical
2019	DeStasio et al. [18]	To describe how health psychology and neuroeconomics can be mutually informative in the study of preventative health behaviors	Theoretical
2016	Van’t Riet et al. [39]	To examine the validity of the risk-framing hypothesis anew by providing a review of the health message-framing literature	Review
2016	Detweiler-Bedell & Detweiler-Bedell [25]	To explore the application of message framing, regulatory focus, construal level and psychological minds to goal setting and self-regulation, and they illustrate the powerful role of subjectivism in determining the effectiveness of health communication.	Theoretical
2012	Gallagher & Updegraff [44]	To distinguish the outcomes used to assess the persuasive impact of framed messages (attitudes, intentions, or behavior)	Review
2011	Mace & Le Lec [37]	To show that this fatalistic behavior can be explained through prospect theory by modeling this overly pessimistic view of old age as a failure to predict the change in the reference point due to hedonic adaptation	Theoretical
2008	Schwartz et al. [27]	To develop two approaches to reducing disutility by directing the decision maker’s attention to either (actual) past or (expected) future losses that result in shifted reference points, on the basis of PT	Theoretical
2007	Siu [45]	To examine effective message design in the promotion of exercise through PT and other theories	Theoretical
1997	Rothman & Salovey [19]	To consider how health recommendations are framed, focusing on the differences in how message framing is operationalized in formal decision problems and experiments in applied domains To examine the impact of message framing on health-relevant decision To explore if the persuasiveness of a framed recommendation relies on the extent to which the message is accepted or deflected by its recipient	Review

PT: Prospect Theory.

**Table A2:** Systematic data of empirical studies.

Year	Author	Aims	Sample	Methodology	Control Group	Results	Limitations
2022	Neumer et al. [58]	To use a health message intervention to motivate customers to engage in distancing behavior	N_1_ = 206 (M = 32.99, SD = 13.87, age range 14–78) N_2_ = 268 (M = 43.68, SD = 17.14, age range 15–86)	Online and field experiment 2 × 2 (gain-loss framed messages and targeting different beneficiaries)	No-intervention baseline	The intervention was more effective when targeting customers than citizens (Exp. 1–2) Loss-framed messages were more effective than gain-framed ones (Exp. 2) Perceptions of risk/worry statistically mediated the effect of messages targeting self-benefits on distancing intentions and behavior	(1) Loss-frame manipulation represents the worst-case consequence (2) Self-developed shortened items (3) Not assess demographics for the Exp. 2
2021	Reinhardt & Rossman [43]	To investigate the effects of framing on younger and older adults’ reactance arousal, attitudes toward the coronavirus vaccination, vaccination intention, and recognition performance	N = 281 (M = 50.1, SD = 23.5)	Online experiment 2 × 2 (gain-loss frame and participants’ age)	Control variables	Loss framing positively influenced vaccination attitudes and led to stronger vaccination intentions among younger adults, but decreased recognition accuracy No framing effects In older adults	(1) Cross-sectional data (2) Higher predisposition to vaccination in older adults (vulnerability for infectious diseases) (3) Possible socially desirable responding (4) Text-only stimuli
2021	Doerfler et al. [56]	To investigate the effects of message framing and personality in relation to risky decision-making during the COVID-19 crisis	N = 294 (M = 39.01, SD = 13.75, age range: 18–78)	Asian Disease Problem (modified) and Dirty Dozen Scale (personality)	No	Both gain- and loss-framing influenced risk choice in response to COVID-19, with more risk-averse in the loss condition Psychopathy emerged as a significant predictor of risk-taking	(1) 40-year-old instrument (2) Brief measure of the Dark Triad traits (3) Sample limited to US-located participants
2021	Fridman [26]	To explore the association between words related to gains or losses and patients’ choices following physician–patient consultations	N = 208	Analysis of transcribed consultations and pre-post treatment decisions	Control variable	Physicians who recommended immediate cancer treatment for cancer used fewer words related to losses and significantly fewer words related to death from cancer Physicians’ use of loss-related words correlated with recommendations for cancer treatment, and loss words were associated with patients’ choice of treatment	(1) Automated text analysis (2) Focus on “gains” and “losses”, just related to cancer survival or cancer death (3) Only male patients
2019	De Bruijn [54]	To explore the effects of message framing to promote dental hygiene	N = 549 (M = 47.4, SD = 16.1, age range 18–87)	2-weeks online experimental study 2 × 2 (behavioral function (detection or prevention) and message frame (gain or loss))	No Baseline	Participants were more likely to select a mouth rinse product that had a preventive function when that prevention function message emphasized gain-framed information Message frame did not impact choice in the detection function condition	(1) Self-reported post-intervention measurement (2) 20% of participants were excluded (3) Priming task to induce either a general non-behavior specific risk-seeking or averse mindset
2017	Lim & Noh [48]	To examine the effect of message framing on users’ intentions to adopt fitness applications	N = 100 (M = 22.3, age range 18–31)	Laboratory experiment employing a designed fitness app (gain- and loss-framed)	No	Advantage of gain-framed messages over loss-framed messages in increasing user’s intentions to use the app Gain-framed messages on users’ intentions to use the fitness app was mediated through exercise self-efficacy and outcome expectations of exercise	(1) No long-term tracking of the behavioral change (2) One period of data collection (3) Text-based message intervention (4) Exercise limited to simple sit-ups
2017	Vezich et al. [53]	To extend predict real-world behaviors (sunscreen use) from neural activity by making direct links to select theories relevant to persuasion	N = 37 women (M = 20.43, SD = 2.44)	Questionnaires, fMRI and 40 text-based ads promoting sunscreen use	No Control ads	Greater MPFC activity to gain- vs. loss-framed messages, and this activity was associated with behavior Stronger relationship for those who were not previously sunscreen users results reinforce that persuasion occurs in part via self-value integration	
2016	Malhotra et al. [33]	To compare attitudes of community- dwelling older adults and patients with advanced cancer for length and quality of life and assess whether these attitudes change with age	N = 1067 CDOAs N = 320 stage IV cancer patients	Quality-quantity (QQ) questionnaire	No Control for differences in sociodemographic characteristics	Lower proportion of CDOAs (26%) than patients (42%) were relatively more inclined towards length over quality of life. With increasing age, the difference in relative inclination between CDOAs and patients increased	(1) Not representative sample (2) Response rate could not be calculated (3) Possible differences in patients (4) Decisions possibly influenced by recommendations
2016	Burns & Rottman [50]	To examine how evaluations of healthiness change as participants consider eating increasing quantities of fruit and to explore how additional contextual features	N = 55 (M = 21.98) N = 72 (M = 20.6)	A 5 (quantity: 1, 2, 3, 4, 5) × 2 (variety: same, variety) × 5 (fruit type: apple, pear, orange, banana, peach) within-subjects design	No	Health benefits that people assign to eating increasing quantities of fruit seem to increase, but only if eating a variety of fruits throughout the day is considered	(1) Lack of ecological validity (2) Individual differences may interact with the manipulations employed
2016	Lucas et al. [28]	To examine the effect of gain versus loss-framed messaging as well as culturally targeted personal prevention messaging on African Americans’ receptivity to colorectal cancer (CRC) screening	N = 132 African-American sample	Online education module about CRC, and exposition to a gain-framed or loss-framed message about CRC screening (2 × 2 × 2)	Yes	Cultural difference in the effect of message framing on illness screening White Americans were more receptive to CRC screening when exposed to a loss-framed message and African Americans were more receptive when exposed to a gain-framed message	(1) Statistically significant differences were not always observed for the reported health messaging differences (2) Small sample size and specific sociodemographic sample (3) CRC screening behavior was not presently assessed
2014	Van’t Riet et al. [40]	To examine the validity of the risk-framing hypothesis	N_1_ = 282 (M = 23.3, SD = 4.55, age range = 18–53) N_2_ = 542 (M = 31.8, SD = 9.96, age range = 18–75) N_3_ = 672 (M = 44.7, SD = 14.6, age range = 15–82) N_4_ = 679 (M = 44.4, SD = 13.9, age range = 18–79) N_5_ = 80 (M = 21.6, SD = 4.25, age range = 18–49) N_6_ = 125(M = 22.9, SD = 5.94, age range = 18–58)	Six empirical studies on the interaction between perceived risk and message Framing (two different countries and employed framed messages targeting skin cancer prevention and detection, physical activity, breast self-examination and vaccination behavior)	No	No evidence in support of the risk-framing hypothesis	(1) Behavioral intention as primary outcome (weak evidence that framing affects behavioral outcomes differently than attitudinal/intentional outcomes) (2) Some samples are women only
2014	Li et al. [47]	To compare message-framing effects on physical activity (PA) across age and gender groups	N = 111 younger adults (M = 2.31, SD = 3.04, age range = 18–35) N = 100 older adults (M = 71.66, SD = 7.48, age range = >60)	Questionnaires (IPAQ pre-post, Instrumental Activities of Daily Living and Subjective evaluation of the messages), accelerometer during 14 days,	No Manipulation check (demographics)	Significant age-by-gender by-framing interactions predicting self-report and accelerometer- assessed PA Older men may benefit particularly from gain-framed PA promotion messages	(1) Self-report items (2) Limited generalizability (3) Not representative groups in terms of the demographic and health-related variables (4) More physical than social benefits
2013	Mathur et al. [29]	To investigate the effectiveness of health message framing (gain/loss) depending on the nature of advocacy (prevention/detection) and respondents’ implicit theories (entity/incremental)	N_1_ = 68 N_2_ = 93 N_3_ = 251	Exp 1. Two-part experiment randomly assigned to a gain or loss frame condition Exp 2. 2 × 2 (implicit theory × frame) between-subjects experiment Exp 3. 2 × 2 × 2 (implicit theory × frame × advocacy) between-subjects design	No	For detection advocacies, incremental theorists are more persuaded by loss-frames. For prevention advocacies, incremental theorists are more persuaded by gain-frames. For both advocacies (detection and prevention), entity theorists are not differentially influenced by frame Entity theorists are message advocacy sensitive, regardless of the message frame.	
2013	Churchill & Pavey [51]	To explore whether autonomy moderated the effectiveness of gain-framed vs. loss-framed messages encouraging fruit and vegetable consumption	N = 177 (M = 21.46, SD = 5.89, age range = 18–57)	Prospective design involving two waves of data collection Questionnaires (demographics, baseline fruit and vegetable consumption, autonomy, framed messages, BMI)	No	Autonomy moderated the effect of message framing. Gain-framed messages only prompted fruit and vegetable consumption amongst those with high levels of autonomy	(1) Self-report measure
2012	Foster et al. [38]	To test the empirical implications of competing theories about how expectations of outcomes affect utility	N = 13,479 (M = 46.05, SD = 16.70)	6-year survey (demographics, SF-36 and health relevant behaviors scale)	No Baseline	Expecting good health in the future increases happiness now	(1) No direct effect of expected outcomes (2) Not clearly if it is sufficiently controlled for current health (3) Individuals relate their health to their prior expectations or to their actual health in the past
2011	Verlhiac et al. [55]	To examine if preventive-behavior framing and outcomes of action framing moderate behavioral intention to stop smoking when health messages are illustrated by pictures	N = 11 (M = 2.1, SD = 0.65)	2 (Preventive Action: presence vs. absence) × 2 (Outcome Behavior: gain vs. loss) × 2 (Outcome Pictures: healthy mouths vs. unhealthy mouths) between-subjects factorial design and questionnaires (State anxiety, behavioral intention)	Yes	Behavioral intention was higher when pictures of unhealthy mouths were presented, regardless of framing, and when pictures of healthy mouths illustrated the presence of preventive action	(1) External validity issues
2011	Gallagher & Updegraff [44]	To examine the effect of fit between the frame and the type of outcome emphasized in a message on subsequent physical activity	N = 192 sedentary adults (M = 19.0, SD = 1.91, age range 16–35)	2 × 2 (frame and extrinsic or intrinsic outcomes) and questionnaires (18-item Need for Cognition (NC) Scale, Exercise Attitudes, Follow-up Exercise, Past Exercise)	No	The predicted interaction between frame, outcome and NC was found such that a ‘fit’ message promoted somewhat, but not significantly, greater exercise for those with high NC, but a ‘non-fit’ message promoted significantly greater exercise for those with low NC	
2009	Winter et al. [34]	To examine through PT if sicker people evaluate quality of life in future health status more positively, compared to healthier people	N = 230 elderly people (M = 76.8, SD = 5.5, age range 69–95)	YDL questionnaire, ADL and IADL (current physical functioning) and demographics	Yes	Interaction between current health status and health scenario supported the relative acceptability of poor-health prospects to sicker individuals	(1) Relatively healthy sample (2) Cross-sectional study (3) Possible race effect
2008	Latimer et al. [46]	Messages to motivate the practice of physical activity emphasizing the benefits (gains) and the costs of inactivity (losses)	N = 332 sedentary people	Sending framed messages and a mixture of the two, and study of cognitive variables and self-reported physical activity in interviews on three occasions	Yes	Gain and mixed frame messages resulted in higher intentions and greater self-efficacy than the loss frame messages (week 2) Gain messages implied increased physical activity (week 9)	(1) Measurement limitations (2) Sample homogeneity (3) Participant-related factors
2006	Lacey et al. [35]	To look at how patients and non-patients rate descriptions of health conditions that differ in severity	N = 159 lung disease patients (M = 67.5, SD = 11.3, age range 23–90) N = 196 healthy participants (M = 39.9, SD = 13.1, age range 18–83)	Survey materials with lung conditions with different levels of severity and QoL questionnaire	Context and no context condition	Perspective of the raters (i.e., their own current health relative to the health conditions they rated) influences the way they distinguish between different health states that vary in severity	
2005	Lauriola et al. [42]	To examine how personality factors affected both risk-taking in decision-making tasks and in real-world health behaviors	N = 240 (M = 46.99, SD = 19.01, age range 20–80)	Framing experiments 3 × 2 (framing condition × valence) about blood cholesterol level or vitamin consumption level Questionnaires (EPQ-R, BIS-BAS, Barratt Scale, the Multidimensional Health Questionnaire, and Coronary Heart Disease items	No	More risk-taking in the negative risky choice framing valence condition and more negative health status evaluation in the negative attribute-framing valence condition. Impulsiveness, Anxiety, Health Involvement and Health Negative Affect correlated with message effectiveness in the goal-framing task and with the observed risk attitude in the risky choice task	
2003	Apanovitch et al. [30]	To compare the effectiveness of 4 videotaped educational programs designed to motivate HIV testing among low-income, ethnic minority women	N = 480 (M = 32, SD = 8.76, age range 18–50)	Structured interviews pre-post, framed videotaped program, self-reported information (HIV and risk factors)	No	Participants’ perceptions of the certainty of the outcome of an HIV test moderated the effects of framing on self-reported testing behavior 6 months after video exposure. In the certain outcome, those who saw a gain-framed video reported a higher rate of testing than those who saw a loss-framed message.	(1) Self-reported information (2) Increased perception of risk and uncertainty diminished the effects of message framing (3) No control condition
2003	Jones et al. [49]	To study the influence of the source credibility and message framing in the promotion of physical exercise in university students	N = 192 (M = 19.81, SD = 4.05)	Positively or negatively skewed messages and questionnaires to assess the impact on intentions and physical exercise	No	It Is helpful to provide exercise-related information highlighting the benefits to motivate clients to exercise	(1) Homogeneous sample (2) Non-objective techniques (3) Lack of impact of persuasive communication on attitudes towards exercise
2003	Winter et al. [36]	To test PT as a model of preferences for prolonging life under various hypothetical health states	N = 384 older people in shared housing (M = 80.6, SD = 7.0)	QoL (Quality of life) questionnaire	No	Participants with health problems preferred a longer life with poorer health conditions than did healthy participants.	(1) General problems due to the type of sample
2002	Broemer [41]	To test the hypothesis that the degree of experienced ambivalence toward health behaviors moderates the impact of differently framed messages	Exp. 1. N = 80 (M = 24.4,SD = 3.89) Exp. 2. N = 120 (M = 25.2, SD = 3.15) Exp. 3. N = 80 (M = 17.6, SD = 2.65)	Health attitudes survey with two framed conditions and questionnaires (perceived personal risk, perceived relevance of health issue, ambivalence, evaluation of the message, attitudes, cognitive elaboration)	No	Highly ambivalent individuals are more persuaded by negatively framed messages whereas individuals low in ambivalence are more persuaded by positively framed messages	(1) Only male participants (exp.1) (2) Not provide direct evidence that ambivalence determines how much subjective weight is given to different health-related outcomes (3) Role of salient behavioral norms might affect reactions to persuasive appeals
2002	Finney & Iannotti [31]	To evaluate an intervention derived from prospect theory that was designed to increase women’s adherence to recommendation for annual mammography screening	N = 929 (age range 40–69)	1 of 3 reminder letters (positive frame, negative frame, or standard hospital prompt	No	The hypothesis that women with a positive history would be more responsive to negatively framed messages, whereas women with a negative history would be more responsive to positively framed letters was not confirmed	(1) Small sample size (2) Is it possible that someone hadn’t receive the message (3) Previous experience with mammography messages
1999	Detweiler-Bedell et al. [52]	Use PT to predict that messages highlighting potential “gains” should promote prevention behaviors such as sunscreen use best	N = 217 (M = 38.7, age range 18–79)	Experiment to compare the effectiveness of 4 differently framed messages (2 highlighting gains, 2 highlighting losses) to obtain and use sunscreen Questionnaires (attitudes and intentions)	No	People who read either of the 2 gain-framed brochures, compared with those who read either of the 2 loss-framed brochures, were significantly more likely to (a) request sunscreen, (b) intend to repeatedly apply sunscreen while at the beach, and (c) intend to use sunscreen with a sun protection factor of 15 or higher	(1) Brief intervention (2) Restricted nature of primary behavioral measure: requests for sunscreen with an SPF of 15 (3) Not collect long-term data

N: sample size, M: mean; SD: standard deviation.
